# Interactions of juvenile hormone, 20-hydroxyecdysone, developmental genes, and miRNAs during pupal development in *Apis mellifera*

**DOI:** 10.1038/s41598-025-93580-7

**Published:** 2025-03-26

**Authors:** T. S. Depintor, F. C. P. Freitas, N. Hernandes, F. M. F. Nunes, Z. L. P. Simões

**Affiliations:** 1 Department of Genetics, Ribeirão Preto Medical School, University of São Paulo, Ribeirão Preto, SP Brazil; 2 Department of Genetics and Evolution, Center for Biological and Health Sciences, Federal University of São Carlos, São Carlos, SP Brazil; 3 Department of Biochemistry and Tissue Biology, Institute of Biology, University of Campinas, Campinas, SP Brazil; 4 Department of Biology, Faculty of Philosophy, Sciences and Letters at Ribeirão Preto, University of São Paulo, Ribeirão Preto, SP Brazil

**Keywords:** *Usp*, *EcR*, *Kr-h1*, *Chd64*, *InR-2*, *Met*, *Ftz-f1*, *Tai*, Metamorphosis regulation, miR-34 and miR-281, Insect hormones, Non-model organisms, Biological metamorphosis

## Abstract

Insect development is primarily controlled by juvenile hormone (JH) and 20-hydroxyecdysone (20E), which regulate gene cascades leading to changes in phenotype, physiology, and behavior. Besides these hormones, microRNAs play a crucial role in insect development by regulating gene expression at the post-transcriptional level. To advance the molecular understanding of holometabolous developmental events, we investigate the pupal phase in the honeybee, *Apis mellifera*. In this study, we assessed the expression profiles of genes components of JH and 20E cascades – *Usp, ftz-f1, EcR, Met, Chd64, InR-2, Kr-h1* and *Tai* – as well as the microRNAs miRNA-34 and miRNA-281 during pupal development of *A. mellifera*. We then analyzed the impact of JH and 20E treatments on the expression of these developmental genes and their putative regulators, the microRNAs. Overall, the selected genes and miRNAs remained stable or were downregulated following 20E treatment, while treatments with JH, upregulated most of our candidate developmental genes and microRNAs. Notably, the expression profile of *Met*, an intracellular receptor of JH, showed a strong correlation with fluctuations in 20E titers during pupal development. Furthermore, a computational analysis, followed by experimental assays, points to both miR-34 and miR-281 as potential regulators of pupal development in *A. mellifera*. This study paves the way for a better understanding of how JH and 20E hormones interact with developmental genes and microRNAs (miR-34 and miR-281) to regulate pupal development in honeybees, elucidating a piece of this complex network of interactions.

## Introduction

In insects, development is characterized by growth periods intercalated by molts, transitioning from immature stages to adults^1–3^. Holometabolous insects, to which our study model belongs, develop from embryo to adult, passing through larval, pre-pupal, and pupal phases. These developmental stages are achieved through a series of molts, primarily regulated by the levels of juvenile hormone (JH) and 20-hydroxyecdysone (20E)^4,5^.

The hormones JH and 20E regulate insect development by exerting opposing effects at specific developmental stages; while JH prevents premature molting, 20E stimulates it. Together, they regulate the precise timing of metamorphosis^6^ by activating gene cascades that result in phenotypic, physiological, and behavioral changes^1,4,5^.

Among the genes whose expression is triggered by JH and 20E, *ultraspiracle (Usp), ecdysone receptor (EcR), Methoprene-tolerant* (*Met*), *kruppel homolog 1 (Kr-h1), calponin-like (Chd64), insulin-like receptor-like (InR-2)*, *nuclear hormone receptor FTZ-F1 (ftz-f1)* and *Taiman (Tai)* stand out as key regulators of insect development^1,7–12^. USP and ECR are nuclear receptors for 20E, promoting the transcription of 20E target genes^13^. Additionally, USP also plays as JH receptor^14,15^; in *Drosophila melanogaster* and *Apis mellifera* it was characterized as component of the JH transcription factor complex^16^. MET acts as an intracellular receptor for JH, and upon binding to the hormone, it translocates to the nucleus where it associates with transcription factors, such TAI and USP. This transcriptional complex then binds to JH response elements in the promoter regions of JH target genes, regulating their expression^17–21^. Downstream of MET and modulated by JH titers, the zinc finger transcription factor Kr-h1 inhibits precocious metamorphosis and adult morphogenesis^1,22^. Playing in the crosstalk between JH and 20E with role in both hormone pathways, is the calponin-like protein, identified as a chaperone, CHD64^8^. When phosphorylated by PKC, triggered by 20E, CHD64 translocates to the nucleus and participates in the 20E signaling pathway. In the absence of phosphorylation, induced by JH, CHD64 binds to USP in the nucleus, integrating into the JH transcription factor complex, mediating JH signal transduction^8^. The insulin receptor 2 activates the expression of 3-hydroxy-3-methyl-glutaryl-CoA reductase, an enzyme that participates in the JH biosynthesis process^7,23^. Finally, the transcription factor FTZ-F1 has been shown to be essential for *corpora allata* activation, participating in JH biosynthesis in *Blattella germanica*^24^. Moreover, it acts as a competence factor at the end of each molt, as a consequence of the 20E peak^25^, allowing expression of downstream genes involved in developmental events (e.g., cuticular genes in pharate-adult stages).

In addition to protein-coding genes, microRNAs (miRNAs) are regulatory non-coding RNAs known to modulate biological processes, including metamorphosis^26–29^. In *A. mellifera*, 197 miRNAs are expressed in the wing discs during metamorphosis (L5-PP2 stage), including miR-281 and miR-34^30^. The miR-281 regulates *EcR* levels during the development of Malpighian tubules in *Bombyx mori* and in a cross-species interaction between the endoparasitoid *Cotesia vestalis* and the moth *Plutella xylostella*. In this latter case, miR-281 is synthesized by *C. vestalis* and modulates *EcR* expression, thereby inhibiting the growth of its host, *P. xylostella*. This highlights the conserved role of miR-281 in targeting *EcR* transcripts across species^31,32^. In honey bees, the expression levels of miR-34 are perturbed by the knockdown of *EcR*^33^.

In this study, we examined the expression profile of the genes *Usp, EcR, Met, Kr-h1, Chd64, InR-2*, *ftz-f1*, and *Tai*, and how their expression is affected by alterations in JH and 20E levels. We also investigated whether miR-34 and miR-281 can bind to the predicted miRNA binding sites on the 3′ UTR of these developmental genes, and assessed their responsiveness to JH and 20E. Our results show that during the pupal development *Met* responds to 20E treatment, while expression of *Usp* is affected by both JH and 20E treatments. miR-34 binds to the 3′ UTR of *InR-2*, *Chd64*, *Kr-h1*, and *ftz-f1*, while miR-281 targets the 3′ UTR of *EcR* and *ftz-f1*. Both miR-34 and miR-281 are upregulated in response to JH treatment and downregulated in response to 20E treatment. Altogether, our study helps to unveil the complex network of interactions between genes, hormones, and miRNAs that orchestrate the honeybee pupal development.

## Material and methods

### Sampling and age control

Honeybees (*A. mellifera*) larvae and pupae were obtained from the colonies kept in the experimental apiary of the Department of Genetics at the University of São Paulo (Ribeirão Preto, Brazil). To control the age of the samples, a fertilized queen was caged in an empty area of the frame for oviposition, for a period of 6 h. To avoid the effects of circadian rhythms on the gene expression of target genes, we implemented a schedule to cage the queen at different times of the day, ensuring that each specific stage could be collected at 10 AM (Supplementary Figure S1).

### Gene expression profile of the candidate genes during preimaginal development

We collected worker bees at each of the following stages: L5S1, L5S2, L5S3, PP1, PP2, PP3, Pw, Pp, Pdp, Pb, Pbl, Pbm, Pbd and Ne (Supplementary Figure S1). The bees were staged according to the classification by Michelette and Soares^34^. To ensure genetic variability, each stage consisted of three worker bees from distinct colonies. The heads were removed from the samples to exclude the primary sites of morphogenetic hormone production, specifically the *corpora allata* and *corpora cardiaca* glands. The remaining body was used to extract total RNA with TRIzol reagent, according to the manufacturer’s instructions. For each sample, 3 µg of total RNA were incubated with 0.5 unit of DNase I Amplification Grade (Invitrogen) and then proceeded to reverse transcription reactions using 100 units *SuperScript II®* (Invitrogen), and 0.5 µg/µL oligo(dT)_12–18_ (Invitrogen). The resulting cDNA was used as template for qPCR reactions using 10 µL of PowerUp™ Sybr™ Green Master Mix (Applied Biosystems), 0.8 µL of each primer (forward and reverse—10 µM), 6.4 µL of water and 2 µL of cDNA (1:10 dilution). Each biological replicate was processed in three technical replicates. The gene sequences were retrieved from the National Center for Biotechnology Information (NCBI) database (version Amel_HAv3.1^35,36^). Gene annotation was performed using the Artemis platform^37^ to precisely define exon boundaries for primer design (Supplementary Figure S2). The sequences and fragment lengths are listed in Table 1. Amplification was carried out in a StepOnePlus™ Real-Time PCR System (Applied Biosystems) following the conditions: 50 °C for 2 min (UDG activation) and 95 °C for 2 min (enzyme activation), followed by 40 cycles of 95 °C for 15 s and 60 °C for 1 min. The dissociation cycle for each primer pair was: from 95 to 60 °C (15 s each degree), 60 °C (1 min), from 60 to 95 °C (15 s each degree).

**Table 1:** Primers used for gene expression analysis of larvae and pupae of *Apis mellifera*.

Gene	Sequence	Length (bp)	Access number (NCBI)
Usp *	F 5′ CTCATCAACCCCGGAAACT 3′ R 5′ ACTTCCGGAGAGAGGGTGGT 3′	181	NM_001011634.2
EcR *	F 5′ CCAACAGCAACAACGGCTAC 3′ R 5′ AAAGAGCCAGGCTGCGACAA 3′	118	NM_001098215.2
*Met*	F: 5′ GGAGAACCCATTTATCTACG 3′ R: 5′ TTGTATTATGGCTCTCATGG 3′	180	XM_395005.7
*Kr-h1*	F 5′ GGAAAAACCATATACTTGCGACA 3′ R 5′ TCCATTGTTTTCTTTGAACCAA 3′	144	NM_001242470.1
*Chd64*	F 5′ GACAAAATCTCAATTCAGTC 3′ R 5′ CTAATTACACCCTGTCCAGC 3′	151	XM_392114.6
*InR-2*	F 5′ GGGAAGAACATCGTGAAGGA 3′ R 5′ CATCACGAGCAGCGTGTACT 3′	171	NM_001246667.1
*ftz-f1*	F 5′ GACTGGGCAAGGAATTCTGT 3′ R 5′ CCATTATGAAGCGTAGTCTCA 3′	152	XM_006557392.2
*Tai*	F 5′ TGAGAAGAGCGATCGTTGGG 3′ R 5′ ATCGCTAACCACAGCCTCAC 3′	161	XM_006563113.3
*miR-34*	F 5′ TGGCAGTGTTGTTAGCTGGTTG 3′ R**	45	miRBase
*miR-281*	F 5′ TGTCATGGAGTTGCTCTCTTTGT 3′ R**	45	miRBase

*F* forward primer, *R* reverse primer. The access numbers were obtained at National Center for Biotechnology Information databases.

*Primers for quantifying *Usp* and *EcR* were used from Barchuk et al.^38^ and Mello et al.^33^ respectively. **The reverse primer for the miRNAs corresponds to the one provided by the NCode™ kit, with the sequence not disclosed.

### Effects of hormonal treatments on candidate genes and microRNAs in pupal stages

We selected white-eyed pupae (Pw) for Juvenile Hormone-III (Sigma-Aldrich) treatment and brown-eyed pupae (Pb) for 20-hydroxyecdysone (Sigma-Aldrich) treatment (for details about the selected stages, see Supplementary Figure S3). Three points were sampled: 1 h, 1.5 h, and 24 h after treatment. The Pw group received a topical application of 1 µL of JH III (3 µg/µL) dissolved in acetone, while the control group was treated with 1 µL of acetone. The Pb group was injected with 1 µL of 20E (3 µg/µL) diluted in saline solution, with the control group receiving only saline (Supplementary Material and Methods). The injections were performed between the 3rd and 4th tergites. The pupae were kept in an incubator at 34 °C with 70% humidity. The development was monitored daily and documented using Zeiss Stereo Discovery V12 (Supplementary Figure S4). Five biological replicates were used in this step of the study.

For the miRNAs, total RNA extraction, cDNA synthesis and qPCR, were conducted following steps mentioned in the previous section. For cDNA synthesis of the miRNAs we used the NCode™ miRNA First-Strand cDNA synthesis Kit (Invitrogen). For this, 2.5 µg of total RNA were used.

### Gene expression analysis

For the expression analysis of candidate genes, we tested reference genes as suggested by Lourenço et al.^39^, the most suitable for normalization was *Rpl32* (see Supplementary Figure S5). In the case of miRNA expression analysis, miR-252a was used as the reference gene, as it exhibited the most stable expression profile across the tested conditions. The relative expression of target genes and miRNAs were assessed following the model (2^−∆∆Cq^ method) proposed by Livak and Schmittigen^40^.

### Prediction and test of miRNAs regulation sites in the candidate genes

For prediction of miRNA binding sites on the developmental genes, the 3′ untranslated region (3′UTR) of the genes: *Usp, EcR, Met, Kr-h1, Chd64, InR-2* and *ftz-f1* were retrieved from the NCBI (Amel_HAv3.1, release 104) and the sequences of miR-34 and miR-281 from miRBase^41^. The regulatory elements were searched using RNAhybrid software^42^. Interactions with binding free energy below − 20 kcal/mol and a *p*-value < 0.05 were considered.

To test if the predicted microRNA regulatory elements (MREs) were functional, we used the *Dual-Luciferase Reporter Assay System* (Promega) as described by Cristino et al.^43^. In summary, the MREs were inserted in the 3′UTR of a luciferase coding region yielding a chimeric PSYCHECK 2 (Promega) vector. HEK293T cells were co-transfected with chimeric vector and mimic miRNA for 24 h and left to rest until next day. Cells lysates were used to measure luciferase activity in a *FlexStation 3 Multi-Mode Microplate Reader*. Luciferase activity was compared between treatment and control groups using *t-test* (*p*-value < 0.05): 1) chimeric vector and mimic miRNA; 2) chimeric vector and scrambled miRNA; 3) chimeric vector only. Six biological replicates were used for each condition.

### Statistical analyses

Statistical analyses were conducted using GraphPad Prism software (version 7, GraphPad Software, Inc., San Diego, CA, USA). Differences between the control and treated groups in the hormone treatment experiments were evaluated using a t-test, with significance set at *p* < 0.05. Correlations between gene expression profiles and hormone levels during pupal development were assessed by calculating Pearson’s correlation coefficient.

## Results

### Expression profile of Usp, EcR, Met, Kr-h1, Chd64, InR-2, ftz-f1 and Tai, and correlation with 20E and JH titers

To test if the expression profile of the genes is correlated with the hormones titers (JH titers obtained by Rembold^44^, and 20E titers from Rachinsky et al.^45^ and Pinto et al.^46^) we calculated the Pearson’s correlation coefficient (r), between the expression profile and circulating hormone titers in the hemolymph (Supplementary Table S1). We established a correlation threshold of ± 0.3, considering moderate correlations or stronger^47^. The genes were then categorized based on their responsiveness: JH-responsive or 20E-responsive.

**Fig. 1:**
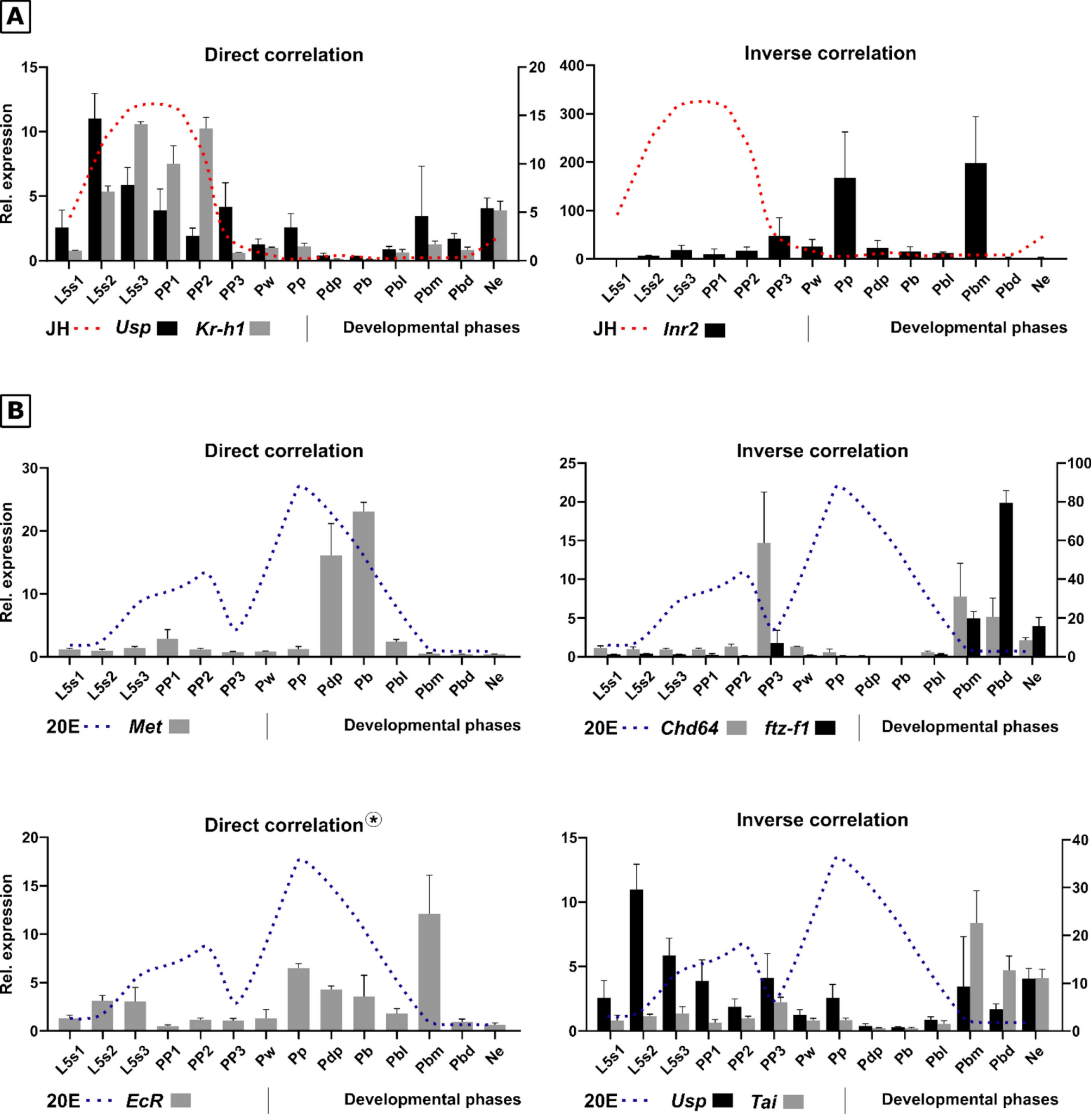
Expression profile of the genes *Usp, EcR, Met, Kr-h1, Chd64, InR-2, ftz-f1* and *Tai* during development of *Apis mellifera* workers, from the fifth larval instar (L5S1) to newly emerged (Ne), and their correlation with Juvenile Hormone (JH) and 20-hydroxyecdysone (20E). (**A**) JH responsive genes: *Usp* and *Kr-h1* exhibit a positive correlation, while *InR-2* shows a negative correlation with hormone fluctuations. (**B**) 20E responsive genes: *EcR* and *Met* shows a positive correlation, while *Usp, Tai, Chd64*, *ftz-f1* and *Tai* exhibit a negative correlation with hormone fluctuations. The JH hormone titers were redrawn from Rembold^44^ and 20E titers redrawn from Rachinsky et al.^45^ and Pinto et al.^46^. Bars show means + -SEM (n = 3, samples processed individually). *  *EcR* showed strong direct correlation with 20E when removing Pbm point (see discussion).

In the set of genes responsive to JH and 20E (Supplementary Table S1), two subclasses are identified: those with direct and inverse correlations. Among the JH-responsive genes, *Kr-h1* (r = 0.9) and *Usp* (r = 0.6) show a direct correlation, where increased hormone titers lead to elevated gene expression (Fig. 1A). In contrast, *InR-2* (r = − 0.31) exhibits an inverse correlation, where high hormone titers inhibit receptor expression (Fig. 1A). Regarding 20E-responsive genes, *EcR* (r = 0.74) and *Met* (r = 0.49) show direct correlations (Fig. 1B), while *Chd64* (r = − 0.48), *ftz-f1* (r = − 0.42), *Tai* (− 0.55) and *Usp* (r = − 0.43) display inverse correlations. Notably, *Usp* presents positive correlation with JH and a negative correlation with 20E during pre-imaginal development.

### Effects of hormonal treatment on developmental genes and microRNAs

**Fig. 2:**
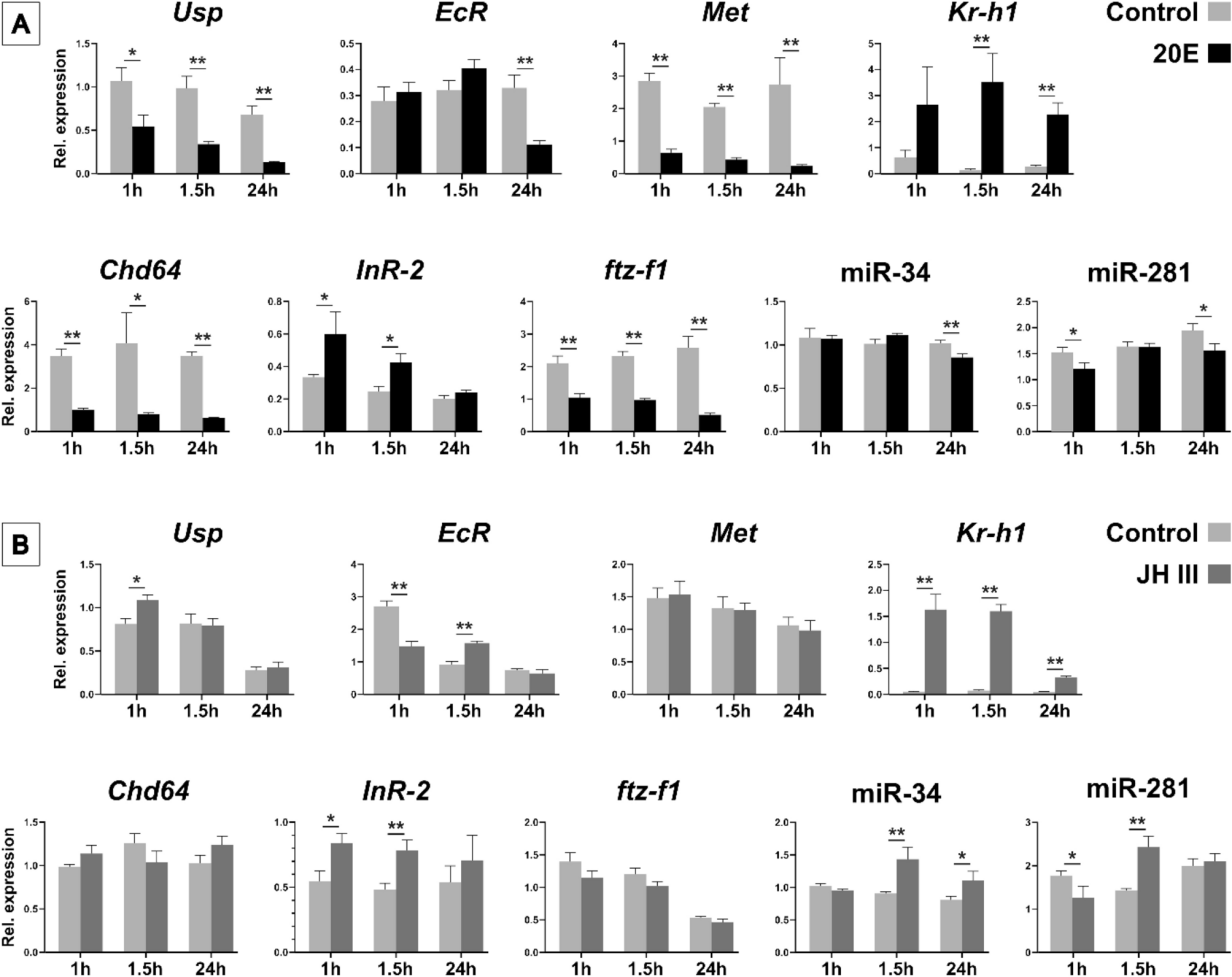
Effects of hormonal treatment on the expression of the genes *Usp*, *EcR*, *Kr-h1*, *Chd64*, *InR-2*, *ftz-f1*, and the miRNAs miR-34 and miR-281 in *Apis mellifera* workers. (**A**) Gene expression in brown-eyed pupae (Pb) treated with 20-hydroxyecdysone (20E). (**B**) Gene expression in white-eyed pupae (Pw) treated with Juvenile Hormone III (JH III). We used 3 µg of each hormone, JH III and 20E, for the treatment of Pw and Pb pupae respectively. The pupae were sampled at 1, 1.5 and 24 h after treatment. Bars show means + -SEM (n = 5, samples processed individually). T-test: * < 0.05; ** < 0.01.

The effects of altered hormonal regimes on developmental genes and miRNAs expression were investigated by qPCR following the application of JH III and 20E on Pw and Pb pupae, respectively (Supplementary Figure S3). The treatment with 20E led to a decrease in the transcript levels of *Usp* (*p* = 0.01), *EcR* (*p* = 0.006), *Met* (*p* = 0.000001), *Chd64* (*p* = 0.02), *ftz-f1* (*p* = 0.003), miR-34 (*p* = 0.007), miR-281 (*p* = 0.03) and an increase of transcripts levels of *Kr-h1* (*p* = 0.007) and *InR-2* (*p* = 0.04) (Fig. 2A). The treatment with JH III increased the transcripts levels of *Usp* (*p* = 0.004), *Kr-h1* (*p* = 0.0003), *InR-2* (*p* = 0.01) and miR-34 (*p* = 0.009) (Fig. 2B). Transcripts levels of both *EcR* (*p* = 0.03) and miR-281 (*p* = 0.04) dropped in JH III-treated pupae 1 h after hormone application, rapidly increased 1h30 after the treatment (*p* = 0.006 and *p* = 0.003) and showed no difference to non-treated pupae 24 h after hormone application.

### Interaction between developmental genes and their regulators miR-34 and miR-281

**Fig. 3:**
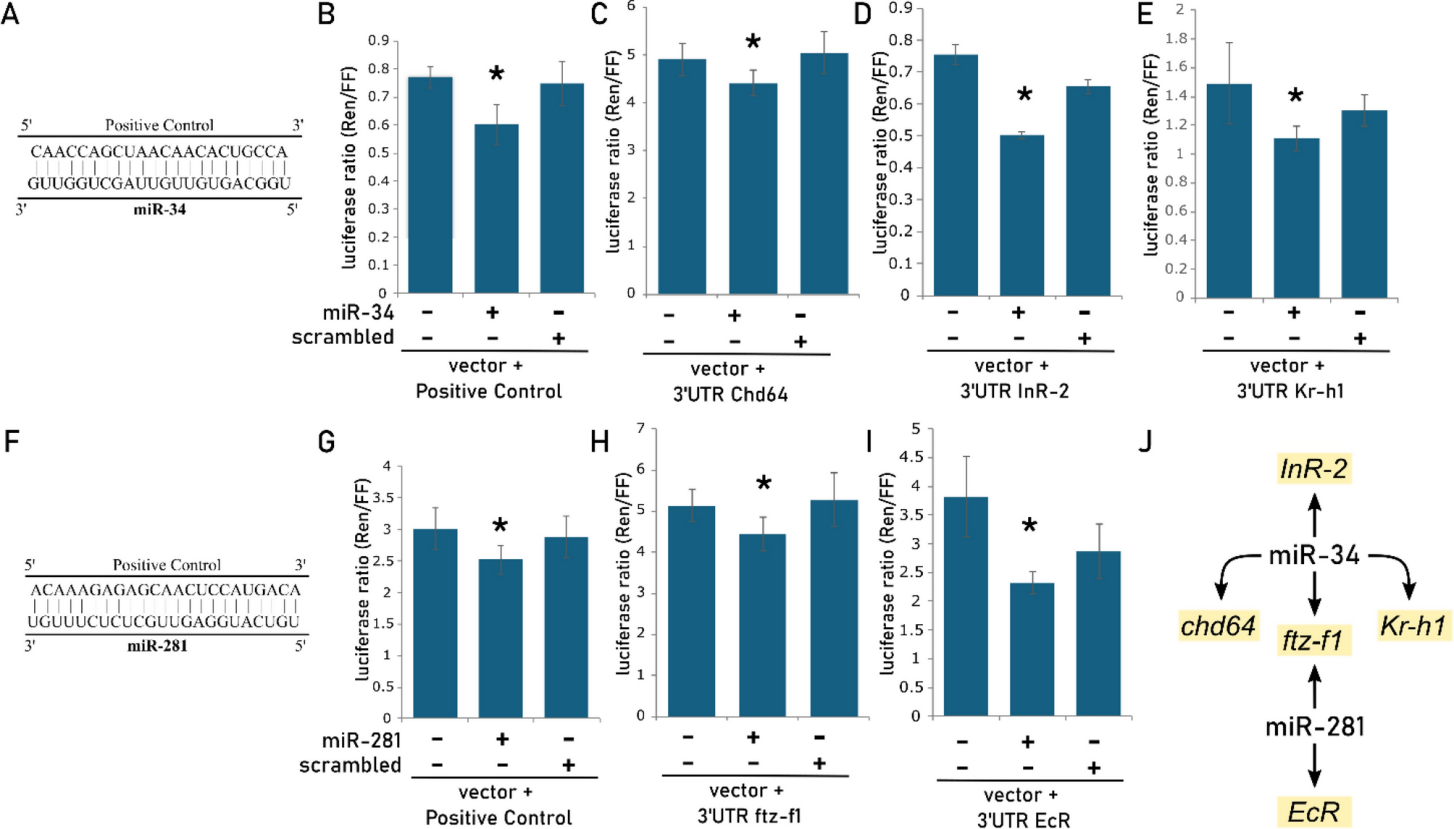
Validation of the predicted binding sites for miR-34 and miR-281 in the 3′UTR of the genes *EcR, Kr-h1*, *Chd64*, *InR-2 and* ftz*-f1,* by luciferase assay. Changes in the ratio of chimeric Renilla luciferase (Ren) and firefly luciferase (FF) activity is shown for each gene. The chimeric Ren luciferase is linked to the 3′UTR of target genes containing the predicted binding sites for miR-34 and miR-281. Schematic representation of the positive control, perfect pairing with the miR-34 and miR-281 sequence (**A** and **F**). The positive control (**B** and **G**) and the 3′UTRs of the *Chd64* (**B**), *InR2* (**C**), *Kr-h1* (**D**) *ftz-f1* (**H**), and *EcR* (**I**) genes were directly regulated by miR-281. (**J**) Network of interactions based on the validated binding sites between the miR-34 and miR-281, in the 3′UTR of the target genes: *EcR, Kr-h1*, *Chd64*, *InR-2 and* ftz*-f1*. Bars show means + -SEM (n = 6) T-test: * < 0.05.

Both JH III and 20E treatments affected the transcript levels of miR-34 and miR-281 (Fig. 2). To further explore the role of these regulatory RNAs, we searched for MREs in the 3′ UTR of developmental genes. MREs for miR-34 were predicted in the 3′ UTRs of the genes *Kr-h1*, *Chd64*, *ftz-f1*, *EcR*, and *USP* (Supplementary Figures S6 and S7), while MREs for miR-281 were identified in the 3′ UTRs of *ftz-f1*, *EcR*, *USP*, and *InR-2* (Supplementary Figure S8). To test if the predicted MREs were functional, we constructed chimeric vectors by inserting the MREs into the 3′ end of the coding sequence of a reporter-gene (Fig. 3A–J). The activity of the reporter-gene was measured following the transfection of HEK293T cells with both the chimeric vector and mimic miRNAs. Mimic miR-34 decreased the gene-reporter activity when co-transfected with chimeric vectors bearing MREs predicted in the 3′ UTR of genes *Chd64* (12.5%) (Fig. 3C), *InR-2* (33.3%) (Fig. 3D) and *Kr-h1* (25.6%) (Fig. 3E). Mimic miR-281 decreased the gene-reporter activity when co-transfected with chimeric vectors containing MREs predicted in the 3′ UTR of genes *ftz-f1* (13.3%) (Fig. 3H) and *EcR* (39%) (Fig. 3I). Based on MREs results, a miRNA-target interaction network was proposed (Fig. 3J).

## Discussion

In *A. mellifera*, the genes *Usp*^38,48–50^*, EcR*^33,51,52^*, InR-2*^53,54^ and *ftz-f1*^55–57^, have been studied in the context of pre-imaginal development. In contrast, research on *Kr-h1,* has focused on the nervous system^58–61^, and for *Chd64* the information available is behavior-related^62–64^.

**Fig. 4:**
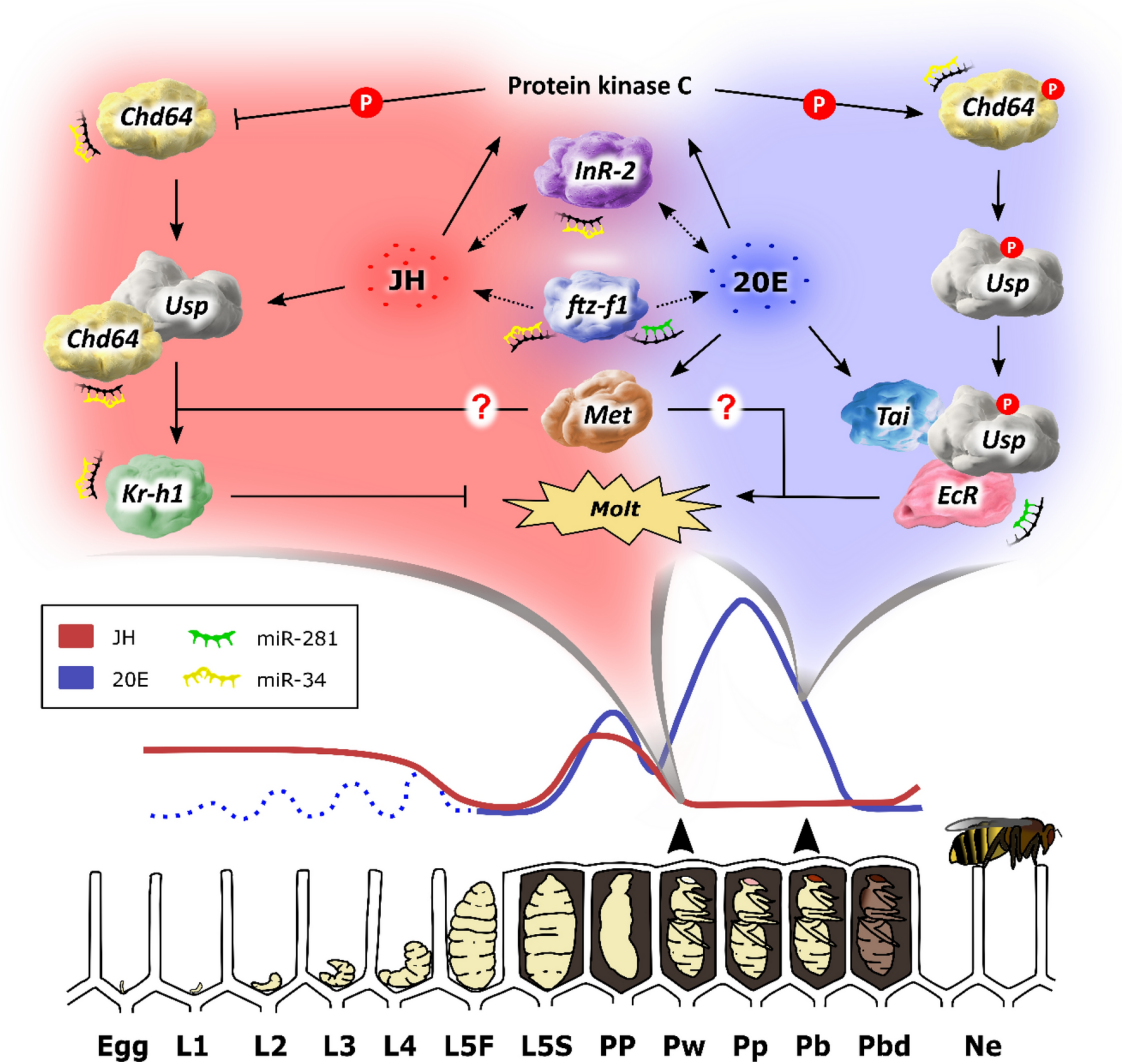
Conceptual model of interactions between the morphogenetic hormones (JH/20E) and the genes *Usp, EcR, Met, Kr-h1, Chd64, InR-2, ftz-f1, Tai*, as well as the miRNAs miR-34 and miR-281 during the pupal development of *Apis mellifera* workers. The modulation of JH titers was obtained from Rembold^44^, while the modulation of 20E titers was based on the studies of Rachinsky et al.^45^ and Pinto et al.^46^. The classification of developmental stages followed Michelette and Soares^34^.

Our results provide a comprehensive analysis of key genes involved in the control of late pre-imaginal development in honeybees (Fig. 4). We show that *Usp* responds to both morphogenetic hormones, and that *Kr-h1* responds very precisely to JH. Meanwhile, *Met*, which is known to participate only in the JH pathway, followed the 20E titers in the assessed stages. The expression of the genes *ftz-f1* and *Chd64* showed to be reliable biomarkers for the metamorphic molt and imaginal molt. Finally, we expand the regulatory network by demonstrating that miR-34 and miR-281 also interact with these genes, pointing to them as regulators of pupal development in honeybees.

### Met expression is affected by 20E and presents stage-specific response to JH-III stimuli

Our results point to a novel role for *Met* in the pupal development of honeybees, independent of the JH pathway, as its expression responds to 20E rather than JH stimuli. Transcript levels of *Met* increase during the pupal stages (Pdp and Pb) in response to the 20E peak at the Pp stage (Fig. 1B), and when 20E levels are maintained high (by injections of 20E in Pb), *Met* expression is also affected. On the other hand, treatment with JH had no effect on *Met* transcript levels during pupal development, when JH levels are basal in honeybees^44^ (Fig. 1B).

*Met* is widely recognized for its role as JH receptor^65–67^ being described in distinct JH-dependent biological processes such reproduction^68^, embryonic development^69^ and development of follicular ephitelial cells^70^. However, its significant expression during the final developmental stages (Fig. 1B), when JH levels are low, raises intriguing questions. In *Tribolium castaneum*, in a study focused on characterizing *Kr-h1* function, high *Met* expression levels can be similarly observed during the pupal phase, when JH is minimal^71^, suggesting *Met* may adopt a JH-independent function at this stage.

We also provide evidence that *Met* responds differentially to JH stimuli depending on the developmental stage. Supplementary Figure S9 offers additional findings, showing that JH III influences *Met* expression in L2 larvae but does not affect *Met* expression in pupal stages, prompting the question: Do tissues lose their competence to respond to JH during pupal development due to the hormone’s sharp decline? At this stage, the organism is preparing for the imaginal molt, making it crucial that no effects from JH interfere with this process. Thus, *Met* is JH-responsive in early stages and 20E-responsive in late development. Bitra and Palli^72^ demonstrated *Met* interacting with ECR and USP in *Drosophila* cell line (two-hybrid interaction assay – L57 cells) while Guo et al.^73^ showed *Met* responding to 20E during pupation in *Bombyx mori*, again by interacting with ECR and USP. Although there is no evidence of interaction between ECR/USP and MET in honeybees, it still supports that MET plays a role in the 20E pathway during pupal development in this species. Additionally, the highest expression levels of *Met* (Pdp and Pb) coincide precisely with a decrease in *Usp* and *EcR* expression levels (Fig. 1A,B), demonstrating that *Met* and *Usp*/*Ec*R have opposing expression profiles in these moments. This indicates a potential interaction—an assertion that requires further experimental validation.

### Usp, a rapid response gene, plays a role in the JH and 20E pathways

USP was identified as component of the ecdysone response complex in 1993^74^. Years later, Jones and Sharp^14^ used fluorescence-based binding assays to demonstrate the USP binding affinity for JH III, indicating a dual role for USP in both hormones pathways. In honeybees, Barchuk et al.^38^ demonstrated that *Usp* expression quickly responds to JH, confirming USP as primary JH binding protein in this species. Our findings show that *Usp* has a positive correlation with JH while displaying a negative correlation with 20E, supporting that during late development of *A. mellifera*, *Usp* plays a role in both hormones signalling pathways. In 2008, Barchuck et al.^49^ first proposed a negative regulation of *Usp* by the morphogenetic hormone 20E in *A. mellifera*, based on expression observations. Our hormonal treatment confirms this, as *Usp* was downregulated by exogenous 20E at all three tested points. This regulatory pattern is consistent across other bee species, the stingless bees *Melipona scutellaris* and *Scaptotrigona depilis*^75^.

The hormonal treatments reveal that while some genes are quickly responsive, others respond more slowly. *Usp* for example responds to JH treatment within 1 h, but its original levels are restored 30 min after that. In contrast, the expression of genes responsive to 20E are not restored even 24 h post-treatment. Barchuk et al.^38^ also observed a rapid response of *Usp* to JH treatment, however, the original expression levels did not restore even 4 h and 30 min post-treatment. The authors used a high dosage of JH III (10 µg/µL), approximately 3.3 times higher than we used here, which probably explains the different results obtained.

Additionally, the expression profile of *Usp* closely resembles that of *Tai*, suggesting a functional partnership. TAI is a highly versatile component involved in regulating both 20E and JH gene cascades. It functions as a steroid coactivator for the 20E receptor complex EcR/USP^76^ and also forms heterodimers with MET in response to JH to repress premature molting^19^. Our results reveal a strong negative correlation between *Tai* expression (r = − 0.55) and 20E titer fluctuations, similar to *Usp* (r = − 0.43), during late honeybee development, suggesting an interaction between TAI and USP in this species. However, our approach focuses on the pupal phase, with only the last larval stages (spinning stages) represented in our data. *Tai* may also correlate with JH, as it mediates JH’s antimetamorphic action through dimerization with MET. However, this effect is expected to occur during the larval molts, and our results do not include this stages.

### Hormonal treatments validate the expression profile correlation

Interactions between hormones and the genes regulating development create a complex and intricate network, where the perturbation of a single component can significantly influence the entire system. Zhang et al.^77^ demonstrated that *Kr-h1*, a well-established target gene for JH, affects the synthesis of 20E by repressing the expression of genes involved in ecdysteroidogenesis in *Drosophila* and *Bombyx*. Given the cross-regulation that exists between hormonal pathways, where one hormone can modulate the effects of another, we correlated the earliest time point of hormonal treatment (1 h post-treatment) with gene expression profile data obtained from specific developmental stages that align with these treatments. For JH III, we analyzed data from the Pw onwards, while for 20E, we focused on the expression data starting from the Pb onwards. This integrated approach enabled us to validate 10 out of the 14 hypothesized relationships presented in this study (see Supplementary Figure S10).

### Kr-h1 and InR-2 have opposite patterns under JH stimuli

The transcriptional regulation of JH target genes, such as *Kr-h*1, is mediated by interaction between USP, MET/GCE and Taiman for insects in general^6,19,78^. The strong positive correlation found between *Kr-h1* expression pattern and JH levels show *Kr-h1* as a reliable biomarker for JH titers in honeybees. Furthermore, this relationship is highly conserved across different insect species, spanning from hemimetabolous^79,80^ to holometabolous insects^71,78^.

The inverse pattern observed between *InR-2* and JH is consistent with the findings of de Azevedo and Hartfelder^54^. This relationship was also identified in *D. melanogaster*, where the insulin-like peptide 6 (DILP6), another component of the insuling pathway, negatively regulates JH titers^81^. However, this poses a contradiction, as *InR-2* levels are low during the feeding stages of *A. mellifera* larvae and peak during the pupal stages, when the organism does not feed. Perez-Hedo et al.^82^ demonstrated that during starvation in *Aedes aegypti*, the expression of the insulin receptor increases alongside expression of *Forkhead-box O* (*FoxO*), a terminal transcription factor of the insulin signaling pathway. *FoxO* coordinates developmental timing and tissue differentiation^83,84^ being active during development, especially during pupal development when feeding ceases. The peaks in *InR-2* expression observed at the Pp and Pbm stages (Fig. 1A) can likely be attributed to the activity of *FoxO* during these periods. In another aspect, *FoxO* is also known to regulate 20E biosynthesis^85^ and to play a role in JH degradation^84,86^. Regarding the modulation of these two key morphogenetic hormones, during honeybee development, *FoxO* is probably involved in the metamorphic transition, in which JH levels are decreasing^44^ and the 20E levels are increasing^45,46^, underscoring the critical importance of *FoxO* during these developmental transitions. Despite the characterization of the *FoxO* gene family in the genome of *A. mellifera*^87^, its function in the context of honeybee development remains to be elucidated.

### Expression profile of ftz-f1 and Chd64 indicates metamorphic and imaginal molt

The genes *ftz-f1* and *Chd64* exhibited similar expression patterns in response to 20E stimuli. As shown in their expression profiles (Fig. 1B), an inverse relationship with hormone titers is observed, which is further confirmed by hormonal treatments, where both genes display the same response to both hormonal stimuli (Fig. 2). This inverse correlation between gene expression and hormone levels has also been documented by Mello et al.^57^ for the *ftz-f1* transcription factor, which requires a decay in 20E levels to increase its expression in honey bees, as it does in *D. melanogaster*^88^. This delayed response coincides with the molting process, occurring after the decay of 20E. The stages examined in this study encompass both metamorphic and imaginal molts, specifically the transitions from PP3 to Pw and from Pbd to Ne. Since the expression of *ftz-f1* and *Chd64* peaks during these stages, their expression profiles are biological markers that distinctly indicate the occurrence of metamorphic and imaginal molts in *A. mellifera.*

### EcR expression peaks after 20E titers drop

*EcR* expression peaks immediately after the plummet of 20E titers in the Pbm stage (Fig. 1B). Truman et al.^89^ demonstrated in *Manduca sexta* that a rise and subsequent fall in ecdysteroid titers is a prerequisite to turn tissues responsive to the hormone. Similarly, in honey bees, Mello et al.^33^ showed that exogenous 20E treatments repress *EcR* expression when 20E levels are high, but *EcR* expression increases once 20E levels decline. This dynamic explains the peak in *EcR* expression profile (Pbm stage) we observed after the significant decline in 20E (Fig. 1B). Due to this specific regulatory mechanism between 20E and *EcR*, we did not include the Pbm value (highest 20E peak) when calculating the Pearson’s correlation between the *EcR* expression profile and 20E titers (Supplementary Table S1). The expression profile obtained for *EcR* and its response to 20E treatments aligns with the findings of Mello et al.^33^, with exception to the results observed under JH treatment. However, the dosage and the chosen stage used for JH treatment is different, Mello and colleagues used Pbl, close to the final pupal development, while we aimed at the beginning of pupal development using Pw. In this study, we assessed isoform A of the gene, which predominates during post-embryonic development, while isoform B is more highly expressed during embryonic stages^33^. Additionally, *Apis mellifera ligustica* also exhibits a similar positive correlation between 20E and *EcR*^52^.

### miR-34 and miR-281 are involved in the regulation of developmental genes

The treatment with JH III notably increased the transcript levels of miR-34, along with its downstream targets, *Kr-h1* and *InR-2*. This observation raises the question of whether miR-34 is directly involved in regulating the decrease of *Kr-h1* and *InR-2* or if the increase in transcript levels observed is merely a response to JH III. In a study by Liu and colleagues^29^, the authors demonstrated that miR-34-KO silkworms (with miRNA ablation via the CRISPR/Cas9 system) exhibited a severe developmental delay during the larval stages, highlighting miR-34 as a regulator of larval growth. This regulatory axis seems to be conserved, as in honeybees, in addition to the increase in miR-34 expression induced by JH III treatment (Fig. 2B), we also confirmed interaction between miR-34 and *Kr-h1* (Fig. 3E). Although the relationship between JH III and miR-34 has not been extensively documented in the literature, our findings suggest a regulatory connection. Furthermore, several other miRNAs have been reported with binding sites in the 3′ UTR of *Kr-h1*^22^, such as miR-2 (*B. germanica*)^90^, miR-927 (*D. melanogaster*)^91^, and let-7 and miR-278 (*Locusta migratoria*)^92^, reinforcing the cooperative role of hormones and miRNAs in regulating gene expression.

While establishing evolutionarily and functionally conserved patterns remains a complex task due to the vast diversity of species and their life histories, our data aligns with prior research. MicroRNAs are known to integrate molecular regulatory networks modulating insect developmental timing and stage transitions^93^. The scientific literature provides conclusive evidence of miRNA-hormone interactions associated with body transformations in Hexapoda. In particular, aspects of organismal growth, including cell proliferation and differentiation, are controled by some JH- or 20E-responsive miRNAs as well as by miRNA-mediated activation of JH- or 20E-signaling^94^. An increasing number of microRNAs crucial for the fine-tuned regulation of insect ontogeny have been well documented, comprising (but not limited to) bantam, let-7, iab-8, mir-13a, miR-13b, miR-100, miR-125, miR-14, miR-2, miR-252a, miR-252b, miR-263, miR-278, miR-281, miR-34, miR-71, miR-8, miR-9, miR-927, miR-965^28,93–98^.

Both miR-34 and miR-281 were upregulated by JH and downregulated by 20E treatment in the pupal development of honeybees. The expression data for miR-34 and *EcR* following 20E treatment (Fig. 2A) align with the findings of Mello et al.^33^, which reported a significant downregulation of miR-34 expression in *EcR*-knockdown bees. The highly conserved miR-34 exhibited a similar response to JH and 20E treatments in *D. melanogaster* embryonic cells^96^, reinforcing that the interplay between these hormones and miR-34 is critical for regulating development and supporting the idea of conserved hormonal regulatory mechanism across insect species.

MiR-281 and *EcR* displayed similar response to JH and 20E treatments in the pupal development of honeybees (Fig. 2A,B) and miR-281 directly targets *EcR* transcripts (Fig. 3I,J). In *Bombyx mori*, the expression of miR-281 was downregulated by 20E but not affected by JH treatments. In this species, miR-281 and *EcR* are co-expressed in the Malpighian tubules from larvae to pupation, with miR-281 overexpression downregulating *EcR* protein levels, while miR-281 suppression leads to *EcR* protein upregulation^31^. Our findings reveal a conserved response of miR-34 and miR-281 to variations in JH and 20E levels, and by validating the interactions of these microRNAs with the developmental genes, we add a new layer of complexity to the understanding of hormonal regulation of gene expression, in this context, fine-tuning the pre-imaginal development process. Combining these findings with the presented discussion and evidence from the literature, we propose a model (Fig. 4) that illustrates a potential regulatory mechanism underlying pupal development in honeybees.

Here, we characterized gene expression dynamics during the late development of *A. mellifera*. Notably, an increase in the transcriptional levels of key genes (*Usp, Kr-h1, InR-2, Chd64, ftz-f1* and *Tai*) as development progresses to the later pupal stages, takes place immediately after the decay in 20E titers. Furthermore, a well-established JH receptor, *Met*, appears to assume a distinct role in pupal development, independent of this hormone. The findings regarding the physical interaction between microRNAs and critical developmental genes further advance our understanding of the relationship between these complexes and the two primary hormones, 20E and JH, which govern pupal progression.

## Supplementary Information

Supplementary Information.

## Data Availability

All data generated or analysed during this study are included in this published article and its supplementary information files.
