# Supplementary material for: Interactions of juvenile hormone, 20-hydroxyecdysone, developmental genes, and miRNAs during pupal development in Apis mellifera
Source: Sci Rep. 2025 Mar 26;15:10354. doi: 10.1038/s41598-025-93580-7 (PMC11937373; doi:10.1038/s41598-025-93580-7)
Supplement: Supplementary file 1 — Supplementary Information. [file 41598_2025_93580_MOESM1_ESM.docx]

**Supplementary Material**

Manuscript: **Interactions of JH, 20E, developmental genes, and miRNAs during *Apis mellifera* pupal development**

Authors: Depintor, TS^1^*; Freitas, FCP^1,3^; Hernandes, N^1^; Nunes FMF^1,2^; Simões, ZLP^1,4^

*E-mail for correspondence: Thiago_depintor@alumni.usp.br


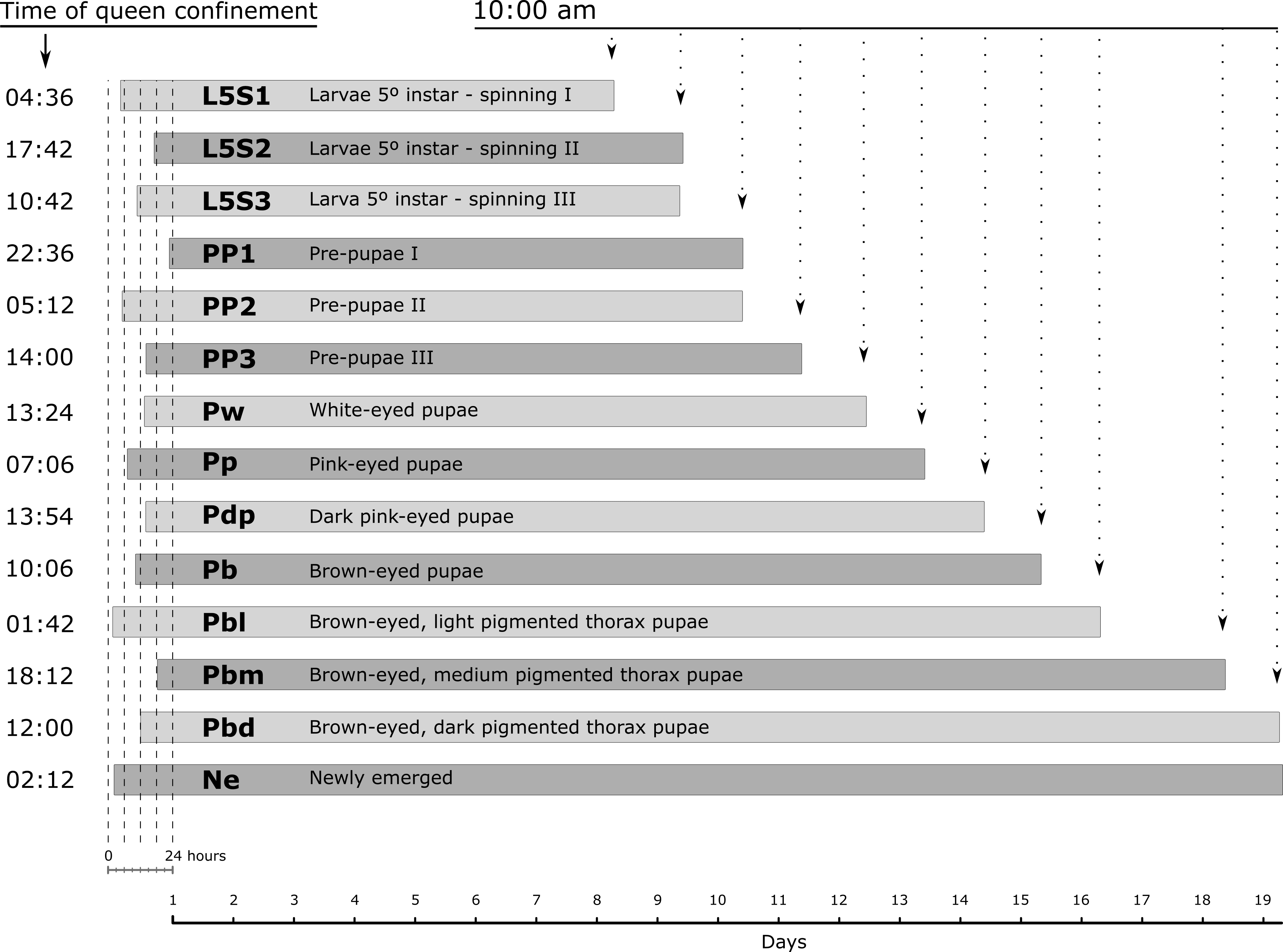


**Supplementary Figure S1.** Representative diagram of the sampling process during the late pre-imaginal development of *Apis mellifera*. In the diagram (left side), the exact time of day when the queen was confined is shown for each of the following points: L5S1, L5S2, L5S3, PP1, PP2, PP3, Pw, Pp, Pdp, Pb, Pbl, Pbm, Pbd, and Ne. To exclude variations caused by hormonal fluctuations throughout the day (reviewed by Di Cara & King-Jones, 2013), we decided to collect all individuals at the same time of day, 10 AM. The calculations and the classification of each stage were based on Michelette & Soares (1993).


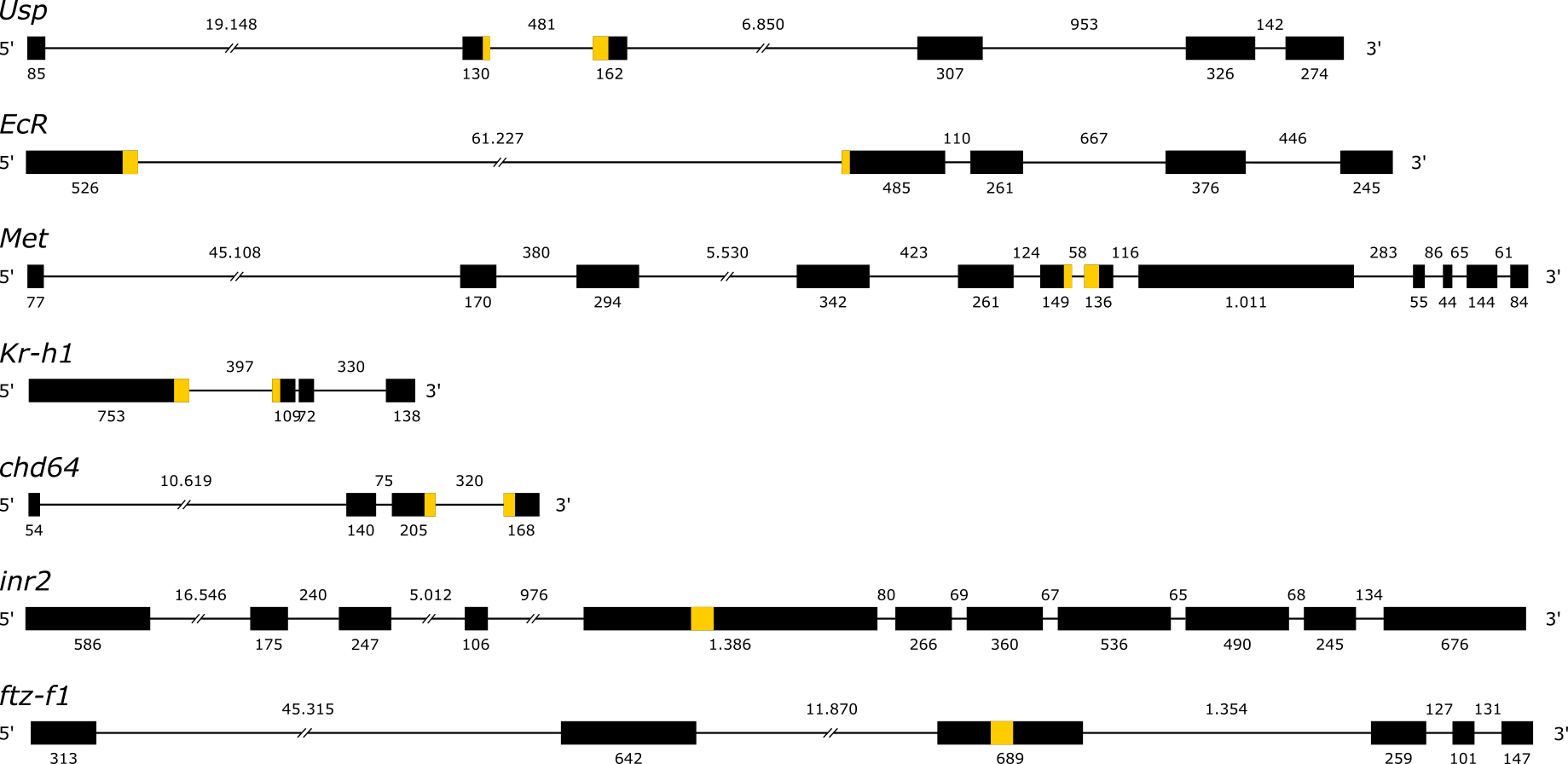


**Supplementary Figure S2.** Annotation of the genes Usp, EcR, Kr-h1, Chd64, InR-2, and ftz-f1 in the genome of Apis mellifera. The black boxes represent exons, while the yellow boxes represent the targeted regions by the primers designed for qPCR, the black solid lines represent introns. Some introns, due to their large size, are abbreviated with two parallel diagonal slashes. The fragment size is indicated above each intron and below each exon. All genes are represented in the 5’ – 3’ direction.


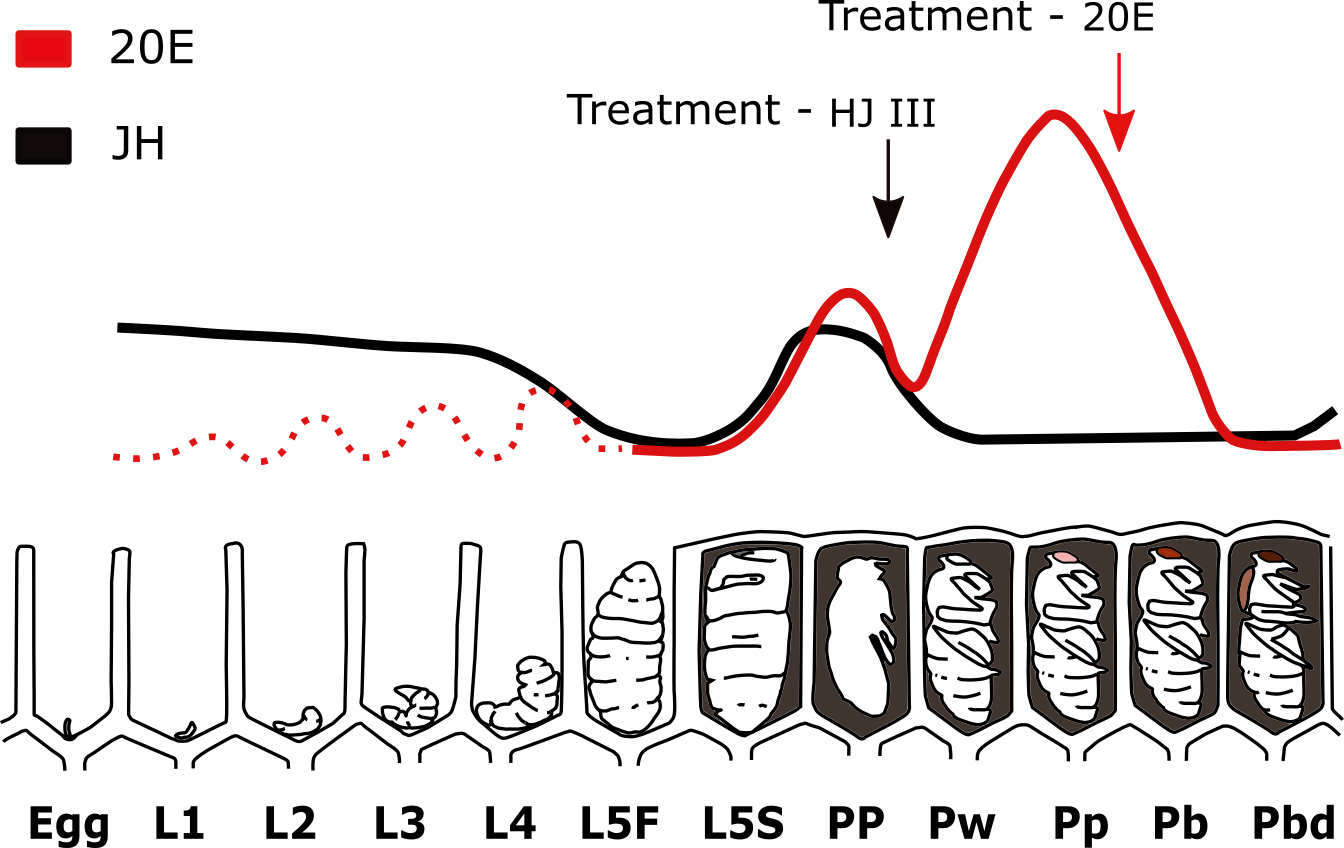


**Supplementary Figure S3.** Moment of the hormonal treatments performed, based on the fluctuations in Juvenile Hormone and 20-hydroxyecdysone titers during *Apis mellifera* development. We aimed to do the hormonal treatments during the pupal phase, once we aimed at the late development. Our idea was to choose a stage in which the hormonal fluctuation was about to decrease for both tested hormones. We believe keeping high levels of the hormone would come closer to a “natural event” than inducing a new peak.


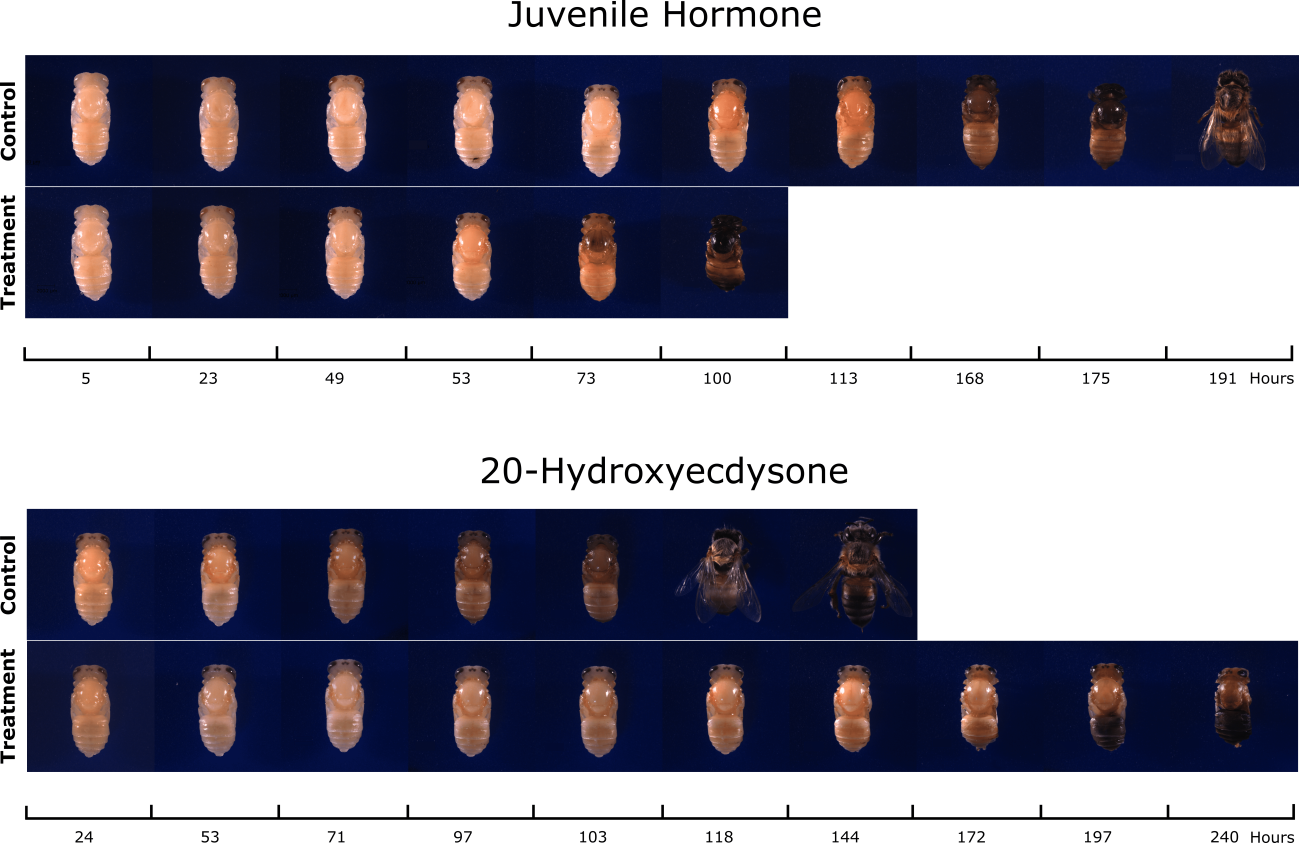


**Supplementary Figure S4.** Validation of Juvenile Hormone (JH) and 20-hydroxyecdysone (20E) treatments during pupal development of Apis mellifera workers. The figure illustrates how treatment with JH accelerates development, in contrast to 20E treatment, which delays development.


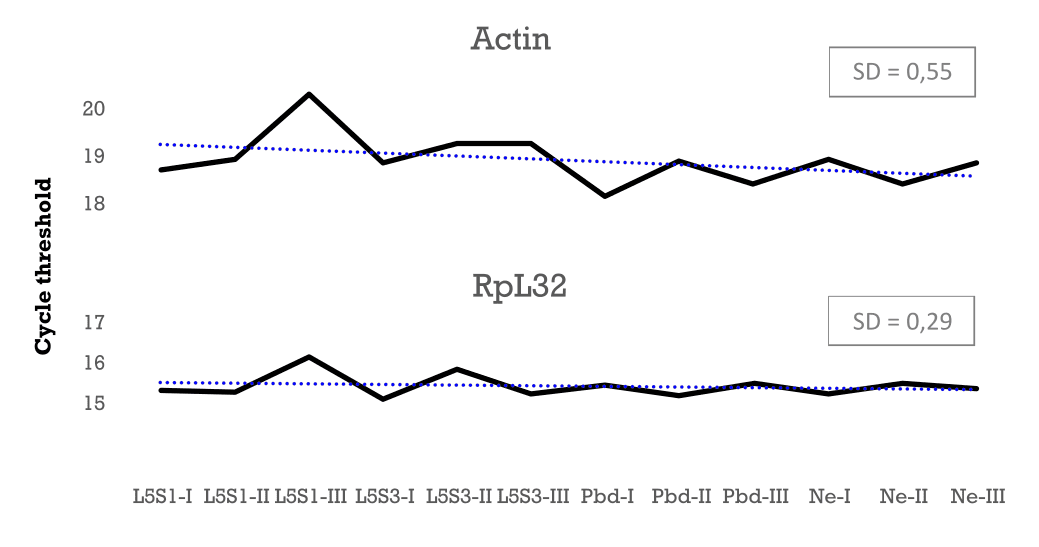


**Supplementary Figure S5*.*** Test of variation across the stages L5S1-I, L5S1-II, L5S1-III, L5S3-I, L5S3-II, L5S3-III, Pbd-I, Pbd-II, Pbd-III, Ne-I, Ne-II, and Ne-III for the genes *RPL32* and *Actin*. The standard deviation (SD) between the points for each gene is represented just above the profile of each normalizer. This testing of variation of reference genes is as suggested by Lourenco et al. (2008).

*Testing variation throughout the stages: L5S1 I, L5S1 II, L5S1 III, L5S3 I, L5S3 II, L5S3 III, Pbd I, Pbd II, Pbd III, Ne I, Ne II and Ne III, for the genes RPL32 and Actin. The standard deviation (SD) between each point is showed just above the profile of each gene.*


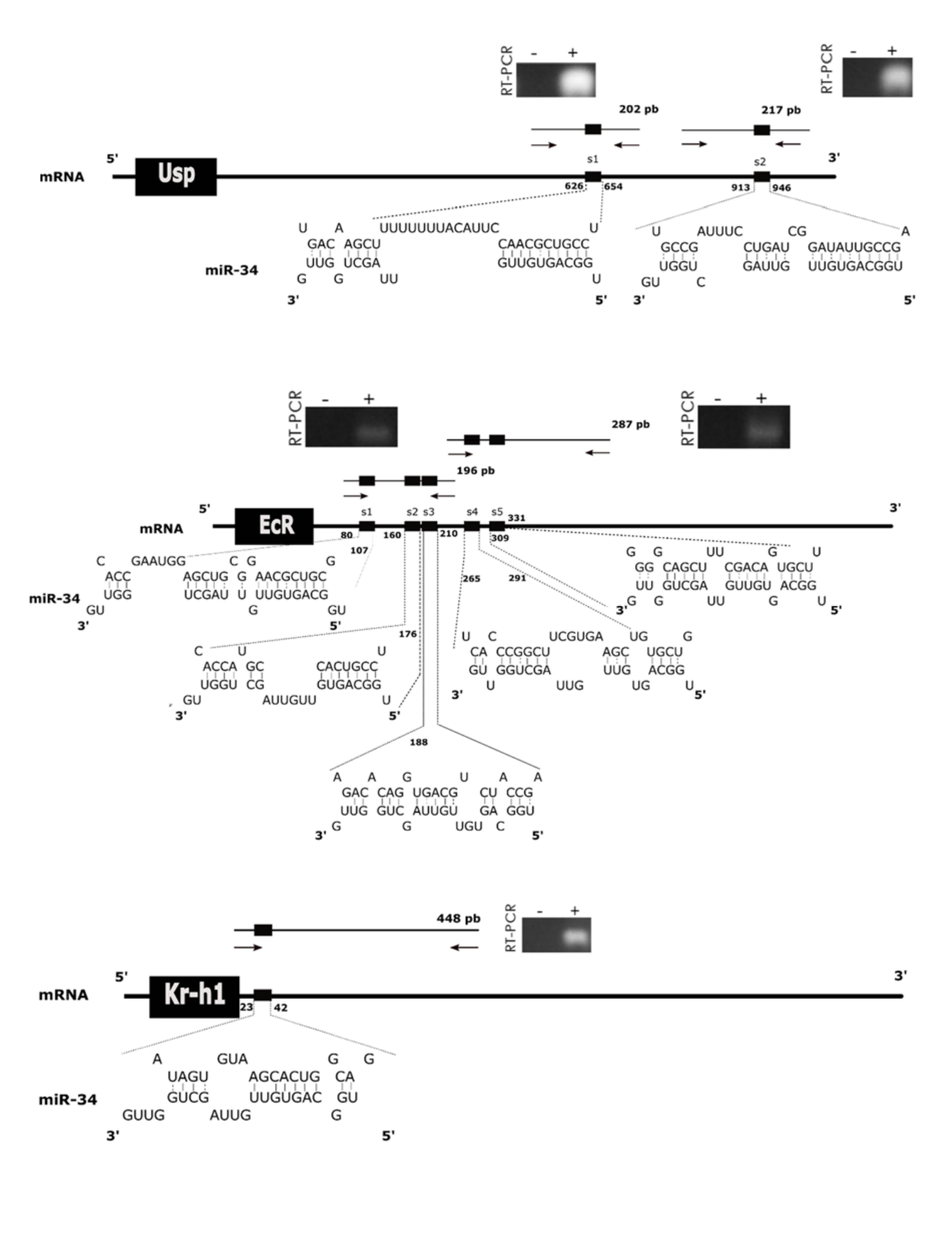


**Supplementary Figure S6.** Predicted interaction sites of miR-34 in the 3’ untranslated region (3’UTR) of the mRNA of the genes *Usp, EcR,* and *Kr-h1* in the genome of *Apis mellifera*. Each graph represents the gene coding region (black rectangle) followed by the 3’UTR (solid black line) and the relative mapped positions where miR-34 is likely to bind. Below each mRNA, a closer view is shown, highlighting details such as molecular structure and its bases.


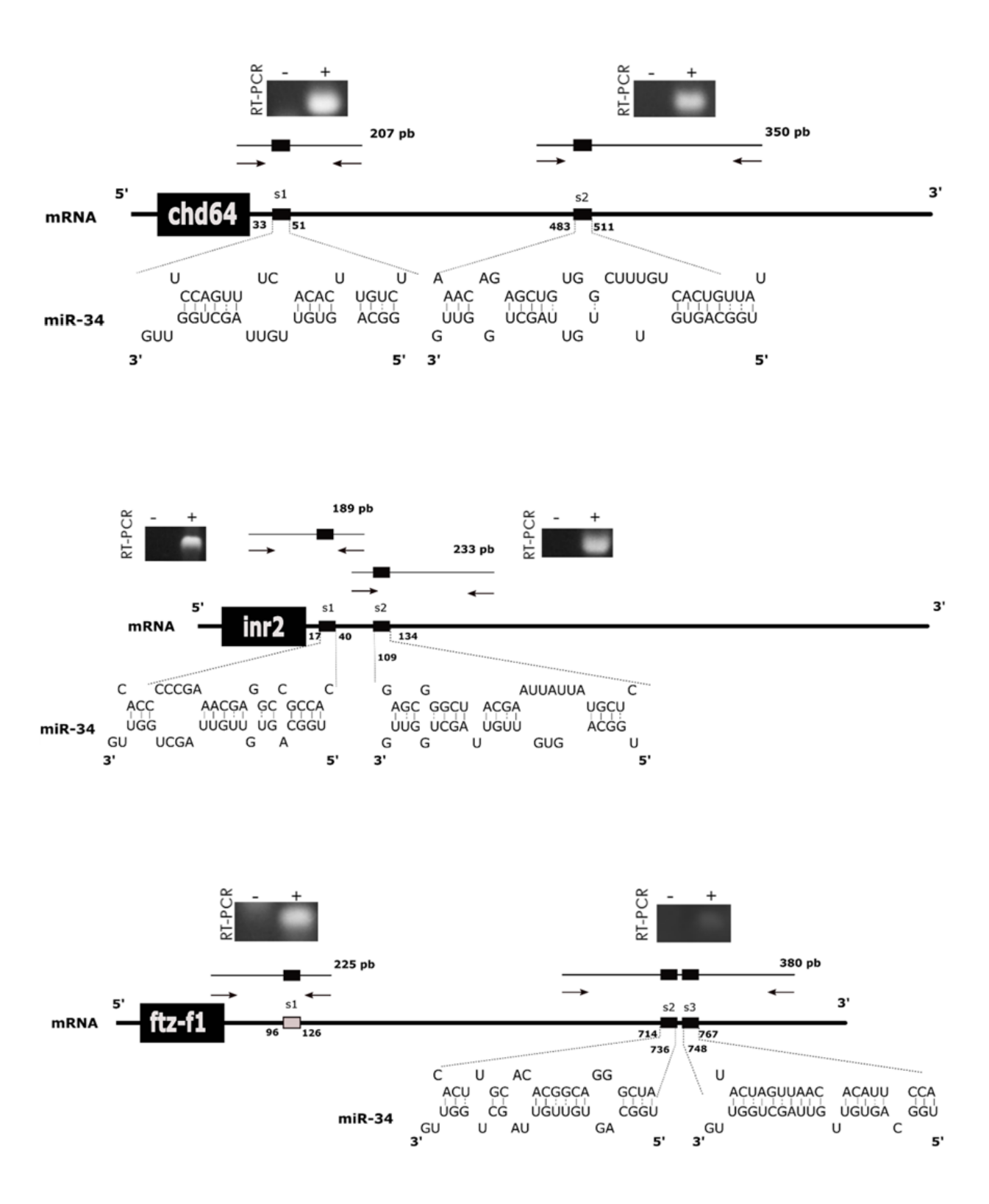


**Supplementary Figure S7.** Predicted interactions sites of the miR-34 in the 3’UTR of the genes: Chd64, InR-2, and ftz-f1 in the genome of Apis mellifera. Each graph represents the gene coding region (black rectangle) followed by the 3’UTR (solid black line) and the relative mapped positions where miR-34 is likely to bind. Below each mRNA, a closer view is shown, highlighting details such as molecular structure and its bases.


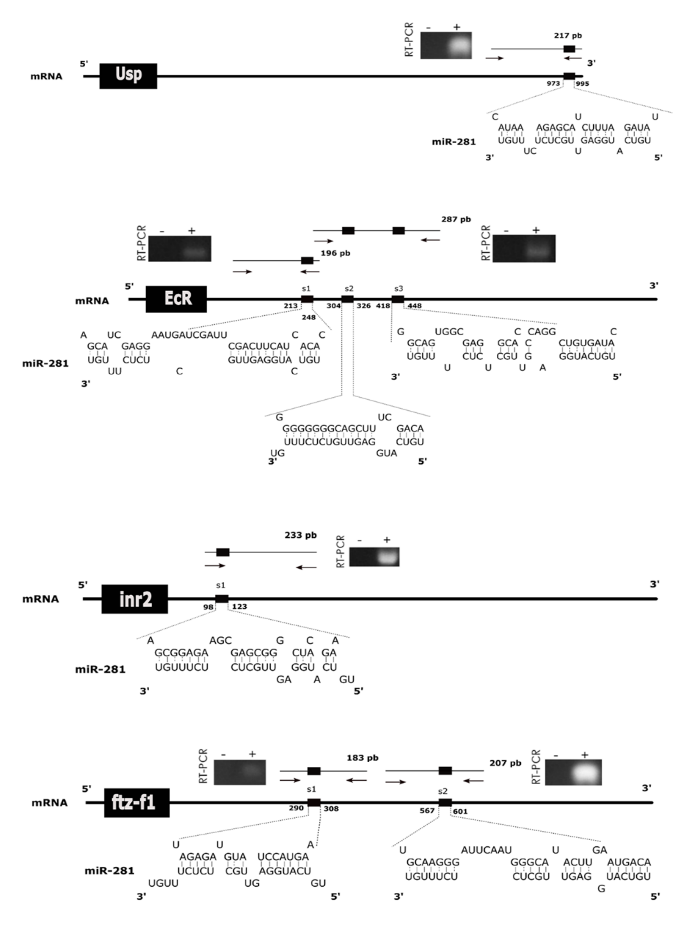


**Supplementary Figure S8.** Predicted sites of interaction between the miRNA-281 in the 3’UTR of the genes: Usp, EcR, InR-2 and ftz-f1 in the genome of Apis mellifera. Each graph represents the gene coding region (black rectangle) followed by the 3’UTR (solid black line) and the relative mapped positions where miR-281 is likely to bind. Below each mRNA, a closer view is shown, highlighting details such as molecular structure and its bases.


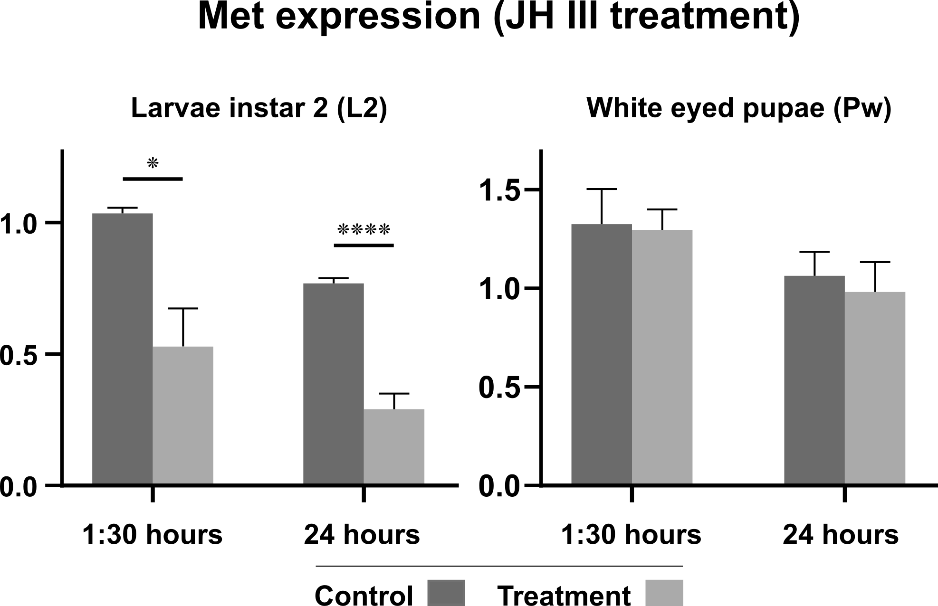


**Supplementary Figure S9.** Methoprene tolerant relative expression under hormonal treatment in instar 2 of larvae (L2) and white-eyed pupae (Pw) of Apis mellifera. We treated L2 (n = 5) with 0,05µg and Pw with 3µg of JH III, the control groups were treated with pure acetone, two points were sampled, 1:30 and 24 hours after treatment. P-value * = 0.01, **** < 0.0001.


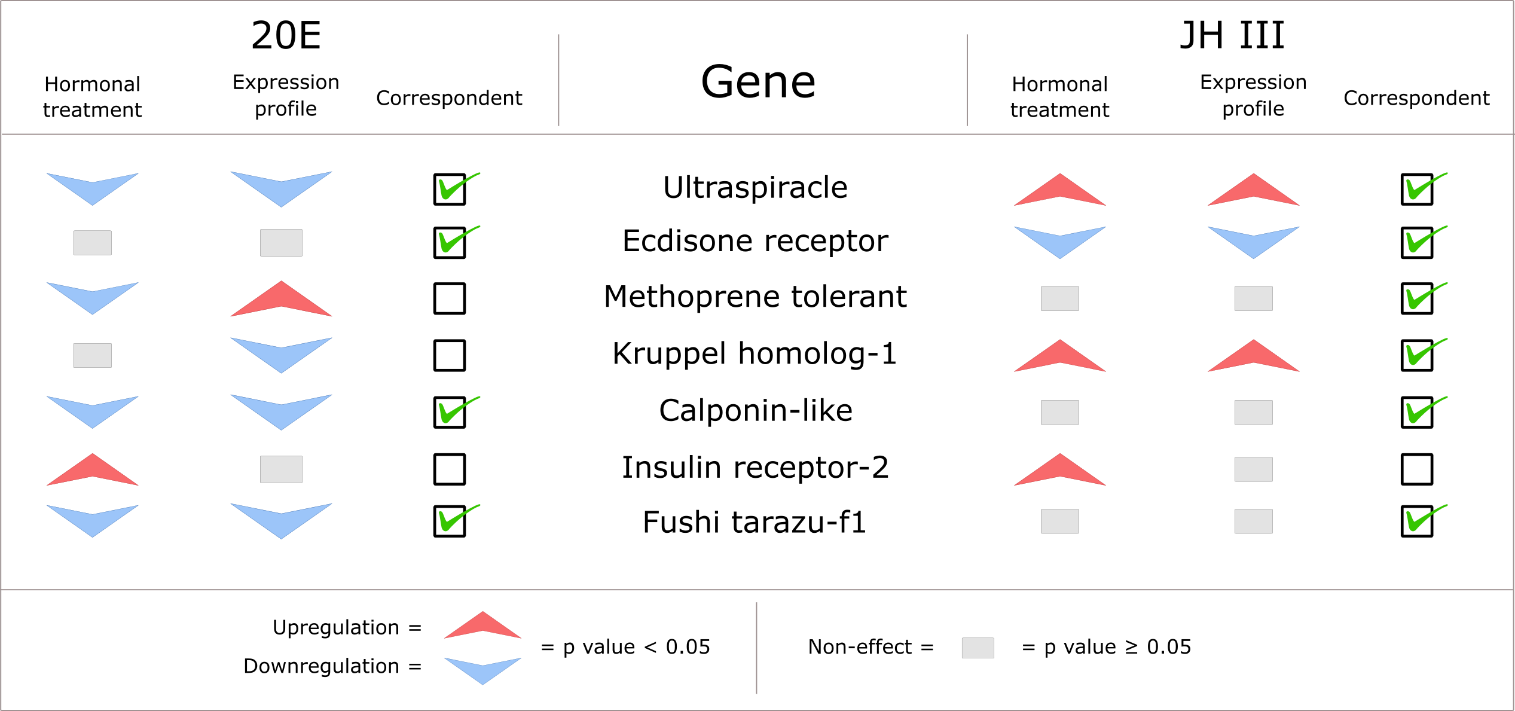


**Supplementary Figure S10*.*** Correspondence between specific pupal stage, with exogenous hormonal treatments (Pw = JH III/ Pb = 20E). The figure summarizes the correspondence between expression profile and the hormonal treatments performed.

Considering the crossed regulation between 20E and JH, in which one path may influence the other, we decided to correlated only the first point of hormonal treatment (1 hour) with expression data from specific developmental stages corresponding to those used in the hormonal treatments (JH III = from Pw on, 20E = from Pb on). So we were able to validate 10 out of 14 relationships (7 genes x2 hormones) between hormonal treatments and expression profile, in which 4 genes matches the profile with 20E treatment (*Usp, EcR, Chd64* and *ftz-f1*) and 6 genes matches the profile with JH treatment (*Usp, EcR, Met, Kr-h1, Chd64* and *ftz-f1*).

**Supplementary Material and Methods**

- JH III was directly diluted in acetone and used in a final concentration of 3 µg/µl.
- 20-hydroxyecdysone (20E) was used from a concentrated sock solution; the stock solution was first be diluted in ethanol and then further diluted into a working solution. Initially, we diluted 2.5 mg of 20E in 125 µl of ethanol (Merck), resulting in a stock solution with a concentration of 20 µg/µl. Next, we added 1.5 µl of the stock solution to 8.5 µl of saline solution (0.9%), creating our working solution with a final concentration of 3 µg/µl.

_________________________________________________________

**Supplementary Table S1.** Correlation between each gene expression profile and titers of JH and 20E in *Apis mellifera larval and pupal development*. * = The *EcR* correlation were measured excluding the Pbm point (see discussion).

| **Pearson correlation coefficient** | | |
| --- | --- | --- |
| **Gene** | **JH** | **20E** |
| *Usp* | 0.601592 | -0.43773 |
| *EcR* | -0.26645 | 0.74265* |
| *Met* | -0.25871 | 0.49255 |
| *Kr-h1* | 0.9039 | -0.0819 |
| *Chd64* | -0.17795 | -0.48456 |
| *InR-2* | -0.31325 | 0.246144 |
| *ftz-f1* | -0.28413 | -0.42398 |
| *Tai* | -0.26411 | -0.55634 |
